# Synchronous restoration of intervertebral disc structure and biological function utilizing cartilage microtissue-based active hydrogels

**DOI:** 10.3389/fcell.2026.1924192

**Published:** 2026-10-30

**Authors:** Shengwen Yu, Xuyu Zhuang, Chen Zhang, Yujiang Liu, Wei Li, Jingming Zhao, Feng Chang, Bo Wang, Shubin Qi, Luoyang Wang, Cuijie Ji, Xizhong Yang

**Affiliations:** 1 Department of Spine Surgery, Qingdao Traditional Chinese Medicine Hospital, Qingdao Haici Hospital, Qingdao, China; 2 Department of Lmmunology, School of Basic Medicine, Qingdao University, Qingdao, China; 3 Department of General Practice, Qingdao Traditional Chinese Medicine Hospital, Qingdao Haici Hospital, Qingdao, China

**Keywords:** cartilage microtissue, hydrogel, injectable biomaterials, intervertebral disc degeneration, tissue engineering

## Abstract

**Objective:**

Intervertebral disc degeneration (IVDD) constitutes a primary cause of low back pain, with existing clinical interventions often failing to facilitate effective regeneration of degenerated nucleus pulposus tissue. This study seeks to develop an active injectable hydrogel composite system incorporating cartilage microtissues (CM) (CM-Gel) and to assess its potential and underlying mechanisms for repairing degenerated intervertebral discs through two approaches, *in vitro* maintenance of cellular phenotype and *in vivo* molecular regulation.

**Methods:**

CM containing cartilage-specific extracellular matrix (ECM) were generated *in vitro* via three-dimensional culture and subsequently integrated with hydrogel to form CM-Gel. Immunofluorescence double staining was conducted on days 1, 4, and 7 of *in vitro* culture to assess the dynamic expression of ACAN and COL-II. An animal model of intervertebral disc degeneration was established, and tissue samples were collected 4 weeks post-surgery. qRT-PCR was employed to measure the mRNA expression levels of anabolic genes, catabolic genes, and the fibrosis marker gene within the nucleus pulposus tissue. Additionally, the reparative effects were evaluated through histological and imaging analyses.

**Results:**

The expression levels of ACAN and COL-II in CM-Gel increased in a time-dependent manner, indicating progressive maturation of the microtissue phenotype. *In vivo* experiments demonstrated that CM-Gel effectively preserved intervertebral disc height and facilitated ECM regeneration. Furthermore, the expression of ACAN, COL-II, and SOX9 was significantly upregulated in the CM-Gel group, whereas the expression of MMP13 and ADAMTS5, as well as COL-I, was significantly downregulated. These molecular changes suggest that CM-Gel promotes matrix synthesis, inhibits degradation, and mitigates fibrosis.

**Conclusion:**

CM-Gel not only preserves the mature phenotype of CM *in vitro* but also reverses the degenerative microenvironment of intervertebral discs *in vivo*, facilitating structural repair and functional restoration by reestablishing the balance between synthesis and catabolism. This composite system presents a novel strategy with potential for clinical translation in the biological treatment of intervertebral disc degeneration.

## 1 Introduction

Intervertebral disc degeneration (IVDD) constitutes a chronic degenerative condition that exerts considerable socioeconomic and healthcare burdens on a global scale (Taylor and Erwin, 2024; Chen et al., 2024). The pathogenesis of IVDD is multifactorial, characterized by key pathological features such as extracellular matrix (ECM) degradation, proteoglycan loss, and apoptosis or senescence of functional nucleus pulposus cells (NPCs) (Wu et al., 2022a; Jin et al., 2024; Sun et al., 2025). These alterations disrupt the homeostasis of the IVD microenvironment, ultimately leading to structural deterioration, impaired mechanical function, and nerve compression. Despite the high prevalence of IVDD, clinical treatment options remain limited. Current therapeutic modalities, including physical therapy, pharmacological pain management, and surgical intervention, predominantly aim at symptom alleviation and do not facilitate reversal or repair of the degenerated tissue, thereby presenting significant limitations (Samanta et al., 2023; Jin et al., 2025; Silwal et al., 2023). Accordingly, the development of novel therapeutic strategies targeting IVDD has emerged as an urgent priority and a central focus within spinal surgery research.

Tissue engineering and cell therapy have emerged as promising strategies for the regenerative repair of IVDD, with various approaches proposed to address this condition. Among these, cell-based and cell-derived methods have been extensively investigated (Krut et al., 2021; Shi et al., 2024a; Genedy et al., 2024). These approaches typically involve the direct injection of mesenchymal stem cells (MSCs) or their derived exosomes, aiming to reverse the degenerative process through paracrine signaling and the induction of targeted chondrogenic differentiation (Liao et al., 2021; Shi P. et al., 2024; Zhao et al., 2025). However, conventional cell injection techniques encounter several significant challenges: transplanted cells exhibit low survival rates and poor retention due to the harsh microenvironment within the IVD; isolated cells face difficulties in forming mature tissue with specific three-dimensional structures and functional properties. Furthermore, these techniques are unable to provide the mechanical strength necessary to support the vertebral body and maintain the intervertebral space. These limitations substantially hinder the efficacy and reliability of cell therapy. Consequently, tissue-engineered IVD substitutes have garnered considerable research interest. These engineered discs possess mechanical strength and can be tailored to the *in situ* IVDD conditions as living tissues. Nevertheless, several challenges continue to impede the clinical application of this strategy, including poor biocompatibility and mismatched degradation rates of scaffold materials, susceptibility of implanted constructs to inflammatory factors in the local microenvironment that impair ECM synthesis, and the requirement for surgical implantation, which entails significant trauma (Xing et al., 2021; Wang et al., 2024).

Hydrogels are widely utilized materials known for their excellent biocompatibility in various tissue regeneration applications (Tolabi et al., 2023; Tamo et al., 2024). Previous studies have employed hydrogels as injectable scaffold materials for IVD repair, enabling minimally invasive treatment while providing immediate biomechanical support (Gao et al., 2024). However, as biomaterials, hydrogels face challenges in achieving true biological regeneration, with their effects often limited to temporary physical filling. Furthermore, the injected hydrogels may exhibit poor integration with host tissue, increasing the risk of long-term displacement or extrusion. Additionally, the degradation rate of hydrogels is difficult to synchronize with the natural tissue regeneration process, potentially resulting in premature loss of mechanical support. Consequently, the development of injectable IVD substitutes that are bioactive, capable of restoring native tissue structure, and able to maintain mechanical strength over extended periods has become a prominent focus of current research.

The advent of microtissue technology presents a novel strategy to address the challenges previously described (Rojek et al., 2022; Liu et al., 2025). Microtissues are miniature tissue constructs generated *in vitro* under three-dimensional culture conditions from tissue-specific cells, which structurally and functionally recapitulate key features of their source tissues (Xie et al., 2022; Wang et al., 2023). In comparison to single-cell suspensions, cartilage microtissue (CM) preassemble a biomimetic extracellular matrix and establish cell-cell interactions *in vitro*, resulting in the formation of stable and functional microtissues. This configuration enhances their resilience to *in vivo* stress following transplantation, preserves cellular phenotype and function, and facilitates more efficient and stable integration and repair through a mechanism analogous to tissue transplantation (Xia et al., 2022). Although CM have demonstrated considerable potential in articular cartilage repair in recent years, their application in IVD repair remains at an early exploratory stage (Kutaish et al., 2023; Jiang et al., 2025a).

This study proposes and validates a minimally invasive injection therapy utilizing CM combined with GelMA hydrogels (CM-Gel) for the regenerative repair of IVDD. Cartilage cells were cultured *in vitro* to generate miniature CM exhibiting characteristic cartilage-like ECM. Their biocompatibility and capacity for cartilage regeneration *in vitro* were compared with cells and confirmed in nude mice. Subsequently, CM-Gel were transplanted via minimally invasive injection into a rat model of IVDD to further assess their *in situ* integration and biological function. This study aims to develop a functional hydrogels for IVD that exhibits adequate mechanical strength to preserve IVD height and effectively simulates the ECM components of the natural NP. This approach is anticipated to offer an innovative tissue engineering strategy for treating IVDD and to facilitate the crucial translational advancement of microtissue technology toward clinical application. The overall study design is shown in Figure 1.

**FIGURE 1:**
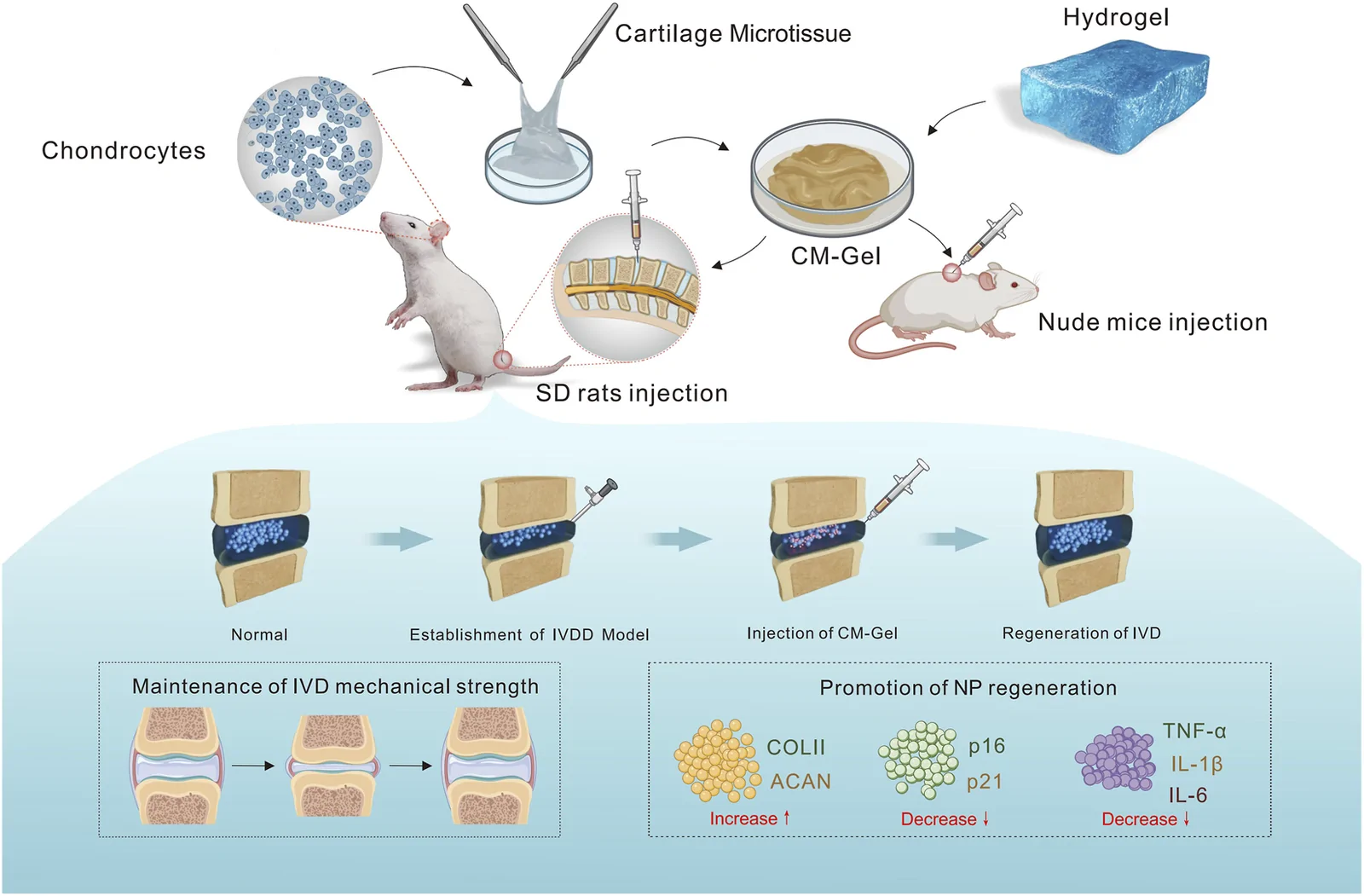
CM-Gel synthesis and its mechanisms in the treatment of IVDD.

## 2 Materials and methods

### 2.1 Cell isolation and culture of chondrocytes

This study received approval from the Ethics Committee of Qingdao Traditional Chinese Medicine Hospital (Qingdao Haici Hospital), approval number is 2025HC01LS060. A total of 48 six-month-old Sprague-Dawley (SD) rats were used in this study. Following anesthesia induced by intravenous administration of 1% pentobarbital sodium at a dosage of 4 mL/kg, cartilage samples measuring 0.5 × 0.5 cm^2^ were excised from the ear and transferred into 50 mL centrifuge tubes containing 0.25% chloramphenicol. Subsequently, the samples were processed within a biosafety cabinet. The ear cartilage was rinsed three times with phosphate-buffered saline (PBS) and eliminated residual fibrous tissue and perichondrium. The tissue was then sectioned into approximately 1 mm^3^ fragments, washed again with PBS, and subjected to digestion with 0.2% collagenase NB4 (Worthington Biochemical Corp., Freehold, New Jersey, United States) at 37 °C for 2 h. The isolated cells were collected and cultured in high-glucose medium (Gibco BRL, Grand Island, New York, United States) supplemented with 10% fetal bovine serum (Gibco BRL) and 1% antibiotic-antimycotic solution (Gibco BRL). Cells were passaged upon reaching over 80% confluence, and third-passage cells were employed for subsequent experiments.

### 2.2 Pre-culture of CM *in vitro*

CM are multicellular microtissues generated by the spontaneous aggregation of chondrocytes within a three-dimensional culture system. These CM effectively recapitulate the cellular composition, spatial architecture, and microenvironmental features of native cartilage *in vitro*. In comparison to conventional two-dimensional monolayer cultures, CM more accurately mimic the cellular interactions and cell-matrix dynamics present *in vivo*, thereby addressing the common issue of chondrocyte dedifferentiation and phenotypic loss observed in two-dimensional culture conditions.

In this study, the harvested chondrocytes were resuspended and seeded into six-well culture plates at a density of 1.5 × 10^7^ cells per well, followed by three-dimensional culture in chondrogenic induction medium. The medium comprised high-glucose DMEM basal medium supplemented with 10 ng/mL TGF-β1 (R&D Systems Inc., Minneapolis, MN, United States), 40 ng/mL dexamethasone (Sigma-Aldrich, St. Louis, MO, United States), 100 ng/mL IGF-I (R&D Systems), 1% insulin-transferrin-selenium-linoleic acid supplement (ITS; ScienCell, California, United States), and 1% antibiotic-antimycotic solution (Gibco BRL). The medium was replaced daily. The medium should be replaced daily. After 7 days, chondrocytes spontaneously aggregate and self-assemble to form microspherical structures exhibiting characteristic histological features of cartilage tissue. These structures constitute the CM utilized in this study. To ensure the reproducibility of CM construction, this study implemented standardized operating procedures. Specifically, the cell seeding density was maintained at 1.5 × 10^7^ cells per well, the culture duration was fixed at 7 days, and the chondrogenic induction medium was refreshed daily at consistent times. Under these conditions, CM produced across multiple batches exhibited high consistency in terms of area, thickness, and histological features. All CM utilized in both *in vitro* and *in vivo* experiments were derived from cultures of at least three independent batches. Hematoxylin and eosin (HE) staining, along with COL-II immunohistochemistry, demonstrated no significant differences in the expression levels of cartilage markers among the different batches.

### 2.3 Preparation of GelMA hydrogel and CM-Gel

To prepare the standard hydrogel, 10% (w/v) GelMA was dissolved in PBS at 50 °C to form the gel precursor, which was subsequently combined with 0.2% (w/v) photoinitiator lithium phenyl-2,4,6-trimethylbenzoylphosphinate (LAP). The prepared GelMA solution was sterilized by filtration through a 0.22 µm membrane filter and stored at 4 °C until further use. Employ a cell scraper to harvest CM, subsequently sectioning the collected cartilage into small blocks measuring approximately 0.1 mm^2^ using scissors. The collected CM was mixed with GelMA at a 1:1 volume ratio to fabricate the CM-Gel construct. Both the pure GelMA and CM-Gel constructs were crosslinked by exposure to ultraviolet light (365 nm, 20 mW/cm^2^) for 5 s.

### 2.4 Characterization of GelMA and CM-Gel

#### 2.4.1 Material characterization

Initially, morphological analysis was performed. Chondrocytes, CM, and CM-Gel samples were examined using an optical microscope (OLYMPUS, CKX41), and images were captured and documented with a Canon DSLR camera (Canon, Japan). Subsequently, at 25 °C, the viscosity and shear-thinning properties of the gel precursors were evaluated using a HAAKE MARS rotational rheometer equipped with a parallel plate geometry (P20 TiL, 20 mm diameter). Shear-thinning behavior was assessed at a 0.5 mm gap over a shear rate range of 0–50 s-1. Dynamic rheological measurements were conducted under 365 nm wavelength light at an intensity of 20 mW/cm2 for 60 s. Time-sweep oscillatory tests were performed at 10% strain (CD mode), 1 Hz frequency, and a 0.5 mm gap for 120 s. The gelation point was defined as the time at which the storage modulus (G′) first surpassed the loss modulus (G″).

Subsequently, swelling ratio measurements were conducted (Gan et al., 2019). The initial volume (V0) and the volume after 24 h (V) of GelMA and CM-Gel constructs were recorded, and the volumetric swelling ratio (SRv) was calculated as V/V0. The radii of the hydrogels before and after swelling were denoted as R0 and R1, respectively, while the initial and 24-h swollen heights were recorded as H0 and H1.

$$\text{SRv}=\frac{\pi R_{1}^{2}\times H_{1}}{\pi R_{0}^{2}\times H_{0}}$$

Enzymatic degradation was assessed through weight measurements. The initial dry weight of the hydrogels was designated as W0, and following immersion in a 0.15% (w/v) collagenase solution, the remaining dry weight was measured and recorded as Wt. The degradation rate was defined as (Wt/W0) × 100%.

Mechanical testing was performed on GelMA and CM-Gel hydrogel disc samples with 10 mm in diameter and 3 mm in thickness using an Instron-5542 universal testing machine (Liu et al., 2024). At room temperature, a compressive load was applied to the crosslinked samples at a loading rate of 0.5 mm/min until the samples underwent 80% deformation relative to their original thickness. Stress-strain curves were recorded, and the compressive modulus was determined by calculating the slope of the linear region of the curves.

#### 2.4.2 Biocompatibility of CM-Gel

After *in vitro* culture for 1, 4, and 7 days, cell viability within the CM-Gel was evaluated using the Live/Dead Viability/Cytotoxicity Kit (Invitrogen, United States) in conjunction with confocal microscopy (Nikon, Japan). Subsequently, the number of dead cells was quantitatively analyzed using ImageJ software. To evaluate chondrocyte proliferation within the CM-Gel, the samples were digested overnight at 56 °C with 250 μg/mL proteinase K to ensure complete DNA release. Subsequently, DNA content in each sample was quantified using the PicoGreen double-stranded DNA quantitation kit (Invitrogen), in accordance with the manufacturer’s standard protocol.

At each designated time point, the samples were washed three times with PBS and subsequently fixed at room temperature using 4% paraformaldehyde for 30 min. Following fixation, the samples were permeabilized with 0.5% Triton X-100 for 15 min and then blocked with 5% bovine serum albumin (BSA) for 1 hour. Primary antibodies targeting ACAN and COL-II were then applied, and the samples were incubated overnight at 4 °C. On the following day, after washing with PBS, the appropriate fluorescent secondary antibodies were added and incubated at room temperature in the dark for 1 hour. Nuclear counterstaining was performed using DAPI for 5 minutes, after which the samples were mounted with an anti-fade mounting medium. Imaging was conducted using a laser confocal microscope under consistent parameters for all samples. Semi-quantitative analysis of ACAN and COL-II fluorescence intensity was performed using ImageJ software, with a minimum of three randomly selected fields analyzed per time point group.

To quantitatively assess the matrix secretion capacity and phenotypic stability of CM cultured within CM-Gel, samples were collected on days 1, 4, and 7 for subsequent ELISA and Real-time fluorescent quantitative PCR (qRT-PCR) analyses. For ELISA, CM-Gel samples collected on days 1, 4, and 7 were thoroughly homogenized. Following centrifugation, the supernatants were collected, and the total protein content of each sample was determined using the BCA assay. Levels of total collagen, glycosaminoglycans (GAG), and type II collagen (COL-II) in the supernatants were quantified using ELISA kits in accordance with the manufacturers’ protocols. Absorbance was measured at 450 nm using a microplate reader, and the content of each analyte were determined based on standard calibration curves. Final concentrations were expressed as the amount of each target protein per Gram of tissue weight.

For qRT-PCR analysis, total RNA was extracted from CM-Gel samples at each time point using TRIzol® reagent and reverse-transcribed into complementary DNA (cDNA). The mRNA expression levels of COL2A1, ACAN, and SOX9 were quantified using the SYBR® Green method, with GAPDH serving as the internal reference gene. Primer sequences are provided in Table 1. Relative gene expression was calculated using the 2^−ΔΔCt^ method, normalizing to the expression levels observed on day 1.

**TABLE 1:** Gene primer sequences utilized for qRT-PCR.

Gene	Forward (5'→3′)	Reverse (5'→3′)
COL2A1	AAT​TTG​GTG​TGG​ACA​TAG​GG	AAG​TAT​TTG​GGT​CCT​TTG​GG
ACAN	AAC​TTC​TTC​GGA​GTG​GGT​GGT	CAG​GCT​CTG​AGA​CAG​TGG​GG
SOX9	GTA​ATC​CGG​GTG​GTC​CTT​CT	GTA​CCC​GCA​CTT​GCA​CAA​C
MMP13	ATG​TGG​AGT​GCC​TGA​TGT​G	AAGCGTGTGCCAGAAGAC
ADAMTS5	TGG​AGT​GTG​TGG​AGG​GGA​TA	CGG​ACT​TTT​ATG​TGG​GTT​GC
COL1A1	GAT​CCT​GCC​GAT​GTC​GCT​AT	GGA​GGT​CTT​GGT​GGT​TTT​GTA​TTC
p16	GTGTGCATGACGTGCGGG	GCA​GTT​CGA​ATC​TGC​ACC​GTA​G
p21	TTG​CAC​TCT​GGT​GTC​TGA​GC	TGC​GCT​TGG​AGT​GAT​AGA​AA
TNF-α	GCA​TGA​TCC​GAG​ATG​TGG​AAC​TG	CGC​CAC​GAG​CAG​GAA​TGA​GAA​G
IL-1β	CAC​CTC​TCA​AGC​AGA​GAC​AG	GGG​TTC​CAT​GGT​GAA​GTC​AAC
IL-6	TCC​TAC​CCC​ACC​TTC​CAA​TGC​TC	TTG​GAT​GGT​CTT​GGT​CCC​TTA​GCC
GAPDH	CCT​CGT​CCC​GAT​GAC​AAA​AAT​G	TGA​AGG​GGT​CGT​TGA​TGG​C

At the same time, we evaluated the ability of CM-Gel to facilitate cartilage regeneration in animal models. CM-Gel was administered via subcutaneous injection into nude mice, and following 4 weeks *in vivo* incubation period, the material was retrieved for subsequent cartilage tissue analysis.

### 2.5 Animal experiment

This study involved a total of 48 male Sprague-Dawley rats, each aged 6 months and weighing between 300 and 350 g. All SD rats were randomly allocated into four experimental groups, each comprising 12 individuals: the Blank group, consisting of untreated normal SD rats; the Control group, which received PBS injections following model establishment; the Gel group, which was treated with GelMA injections post-model establishment; and the CM-Gel group, which received CM-Gel injections after model establishment. For all rats in the CM-Gel group, autologous cartilage tissue was harvested and cultured with CM as previously described. All rats received treatment on a single IVD located at the C5–C6 segments of the tail vertebrae.

With the exception of the blank control group, the remaining three groups of SD rats underwent IVD injury surgery to establish a model of IVD degeneration. All rats were anesthetized with 3% isoflurane prior. Initially, the locations of the intervertebral spaces were identified and marked. Subsequently, an 18-gauge puncture needle was inserted approximately 5 mm into the IVD, rotated 90° clockwise, and maintained in this position for 45s. Subsequently, 10 μL of PBS, GelMA, and CM-Gel were administered into the intervertebral spaces of the respective groups of rats. Both GelMA group and the CM-Gel group were immediately exposed to ultraviolet light (365 nm, 20 mW/cm^2^) at the injection site through the skin for a duration of 5 s to achieve initial *in situ* gelation and fixation following the injection.

All rats were housed under standard conditions following surgery and underwent assessments at 4 and 8 weeks post-operation.

### 2.6 Imaging and measurement

X-ray and magnetic resonance imaging (MRI) analyses were conducted on the rats at 4 and 8 weeks following injection. All rats were anesthetized with 3% isoflurane prior to MRI scanning, which was performed using a Bruker 7 T small animal *in vivo* imaging system (BioSpec 70/20 USR). T2-weighted images were acquired to assess the water content of the NP tissue. ImageJ software was utilized to evaluate the signals of nucleus pulposus tissue, with all measurements normalized by comparison to the Blank group for accurate standardization. X-ray images were acquired with a digital cabinet X-ray machine to evaluate the disc height index (DHI). Subsequently, ImageJ software was employed to quantitatively measure the centrum height, and the was calculated from these measurements. DHI = twice the sum of the anterior, middle, and posterior edge heights of the intervertebral space/the sum of the anterior, middle, and posterior heights of the adjacent vertebral bodies. The results were normalized using the Blank group as the baseline, and the relative maintenance of IVD height in each group was ultimately expressed as a percentage. X-ray imaging was employed to capture images of the tail vertebrae in all animals (n = 6 per group per time point) for the purpose of calculating the DHI. Additionally, MRI scans were conducted on three randomly selected animals per group at each time point.

### 2.7 Histological, immunohistochemical and immunofluorescence analysis

Animals in each group were euthanized at 4 and 8 weeks post-surgery (n = 6 per group per time point), and tail vertebrae tissue samples were collected. For each group and time point, samples from three randomly selected animals were allocated for histological staining and immunohistochemical analysis, whereas samples from the remaining three animals were utilized for qRT-PCR analysis. Samples collected over 8 weeks period were concurrently utilized for immunofluorescence staining analysis. All rats were euthanized via intraperitoneal injection of sodium pentobarbital at a dosage of 150 mg/kg, after which tail vertebrae tissue samples were collected for subsequent analysis. The samples were initially fixed in a 4% paraformaldehyde solution for 3 days, followed by transfer to a 10% ethylenediaminetetraacetic acid (EDTA) decalcifying solution (Servicebio, China) and decalcified at 37 °C. Upon completion of decalcification, the samples were embedded in paraffin, sectioned at a thickness of 5 μm, and mounted on glass slides. The prepared sections were then stained using HE and Safranin-O&Fast-Green (SO-FG) staining methods. As previously indicated, the histological grading system is categorized into four levels, with scores ranging from 4 points, representing normal tissue, to 12 points, indicating severe degeneration (Masuda et al., 2005). Histological scoring was conducted independently by two observers who were blinded to the group assignments. The mean score from both observers for each group was utilized in the statistical analysis.

During the immunohistochemical analysis, the samples were initially treated with 3% hydrogen peroxide for 10 min, followed by blocking with 5% bovine serum albumin (BSA) at room temperature for 30 min. Subsequently, the samples were incubated overnight at 4 °C with mouse anti-rabbit monoclonal antibodies specific for type I collagen (COL-I),COL-II, and aggrecan (ACAN). Thereafter, the samples were incubated with secondary antibodies at room temperature for 1 h. The tissue sections were then developed using a DAB chromogenic reagent. All reagents utilized in this procedure were obtained from SANTA CRUZ Biotechnology, United States.

For immunofluorescence staining, cells were permeabilized using 0.5% Triton X-100 for 20 min, followed by blocking with 5% BSA at room temperature for 1 h. After discarding the blocking solution, the samples were incubated overnight at 4 °C with the primary antibodies: anti-TNF-α, anti-IL-1β, and anti-IL-6. The following day, the samples were washed with phosphate-PBS and incubated with the appropriate fluorescently labeled secondary antibodies at room temperature in the dark for 1 h. Nuclear counterstaining was performed using DAPI for 10 min, after which the samples were mounted with an anti-fade mounting medium prior to image acquisition.

For the semi-quantitative analysis of immunohistochemical staining, five random fields per section were captured using identical imaging parameters. The mean optical density (MOD) of positive staining for ACAN, COL-I, COL-II, TNF-α, IL-1β, and IL-6 was measured with ImageJ software.

### 2.8 Gene expression analysis

Rat caudal IVD tissue was carefully isolated under sterile conditions. The annulus fibrosus was meticulously removed under a microscope to precisely extract the NP tissue. The collected NP samples were promptly placed into Eppendorf tubes containing RNA preservation solution (RNAlater, Invitrogen), stored at 4 °C overnight, and subsequently transferred to −80 °C for long-term storage. qRT-PCR was performed following the protocol detailed in Section 2.4.2. The target genes analyzed included anabolic markers (COL2A1, ACAN, SOX9), a fibrotic marker (COL1A1), catabolic markers (MMP13, ADAMTS5), cellular senescence markers (p16, p21) and pro-inflammatory cytokines (TNF-α, IL-1β, IL-6) with Gapdh serving as the internal reference gene. Primer sequences are detailed in Table 1.

### 2.9 Statistical analysis

In this study, we utilized GraphPad Prism 9.5 software for data analysis and chart creation. The sample size was determined based on the outcomes of a preliminary experiment, which indicated a 25% difference in DHI between the CM-Gel group and the PBS group, with a standard deviation of 15%, an alpha level of 0.05, and a statistical power of 80%. This calculation yielded a minimum requirement of five animals per group at each time point. To account for potential animal mortality or model failure, the sample size was increased to six animals per group per time point. Comparisons among multiple groups were conducted using one-way analysis of variance (ANOVA), followed by pairwise comparisons employing Tukey’s *post hoc* test. A p-value less than 0.05 was considered indicative of statistical significance.

## 3 Results

### 3.1 Preparation and characterization of CM-Gel

As shown in the optical microscope image, P3 chondrocytes display a polygonal morphology, indicative of their viability (Figure 2A). Chondrocytes generate uniformly sized, sheet-like CM at the base of well plates. These CM exhibit a pale white coloration and a soft texture when observed macroscopically, with a smooth surface morphology (Figure 2B). The CM are evenly distributed across the six-well plates, and the area and thickness of the sheets formed demonstrate consistent reproducibility both between different wells and across separate experimental batches. Histological analysis demonstrated that HE staining revealed chondrocytes cultured for 7 days within the CM were localized within characteristic cartilage lacunae. The cells exhibited a round or oval morphology, abundant cytoplasm, and centrally positioned nuclei, all encompassed by a basophilic cartilage matrix (Figure 2E). SO staining demonstrated a strong positive expression of proteoglycans within the CM, evidenced by distinct orange-red staining (Figure 2F). Immunohistochemical staining further confirmed that COL-II was strongly expressed in the CM, with distribution observed around the cells and throughout the entire microtissue (Figure 2G). This result indicates that, following 7 days of *in vitro* culture, the CM has synthesized cartilage-specific extracellular matrix predominantly composed of COL-II and proteoglycans. In subsequent experiments, the harvested CM membrane sheets were sectioned into uniform small fragments of approximately 0.1 mm^2^ using standardized protocols, combined with GelMA at a 1:1 volume ratio, and crosslinked under ultraviolet light to generate the CM-Gel composite (Figures 2C,D).

**FIGURE 2:**
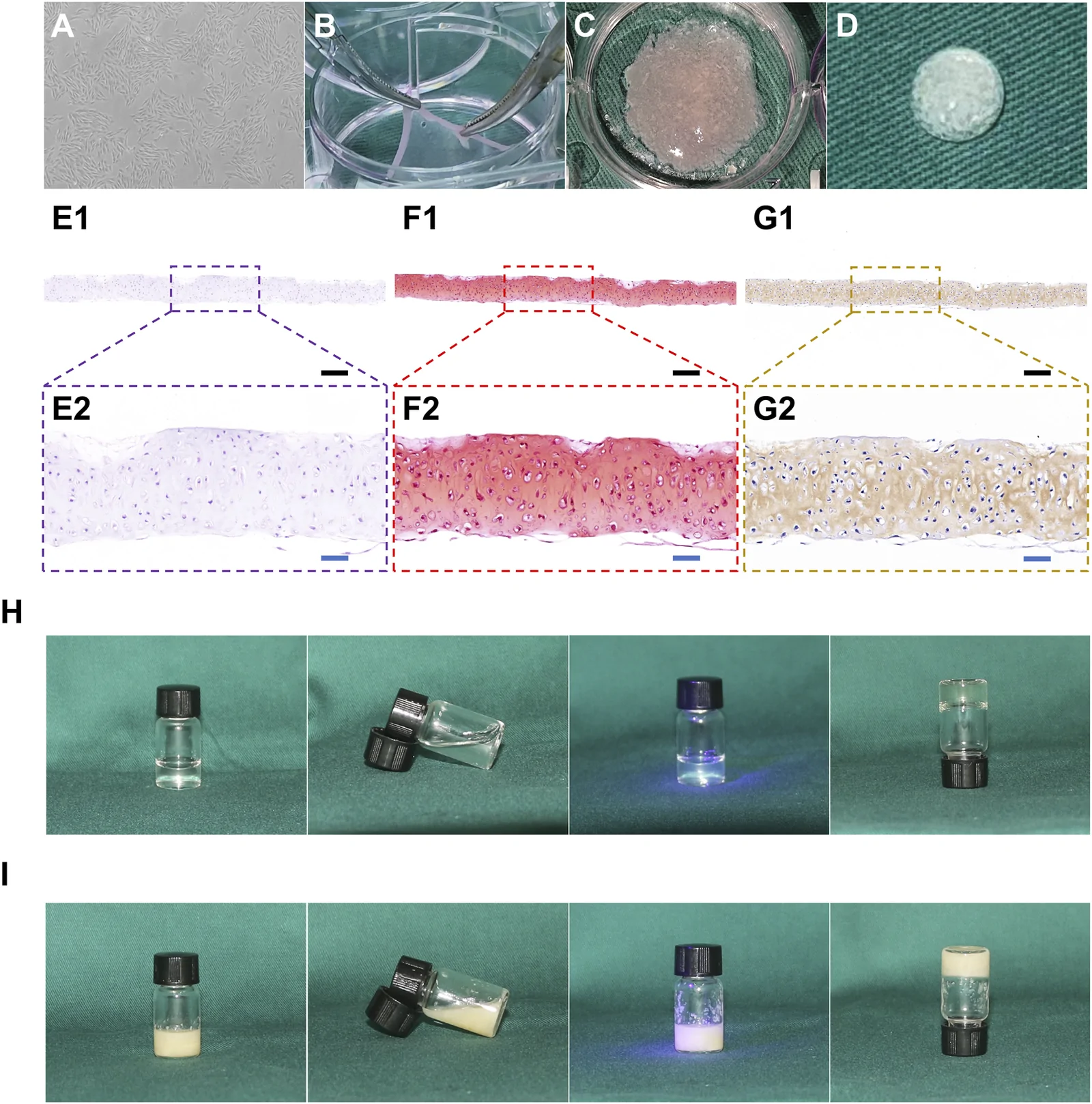
Preparation and characterization of CM and CM-Gel. **(A)** Morphology of P3 chondrocytes observed under an optical microscope. **(B)** Macroscopic appearance of the cultured CM. **(C)** Procedure for preparing the CM-Gel composite by combining CM fragments with GelMA solution. **(D)** Macroscopic appearance of the CM-Gel composite following photo-crosslinking. **(E)** HE staining demonstrating characteristic cartilage lacuna structures within the CM. **(F)** SO staining indicating strong positive expression of proteoglycans within the CM. **(G)** Immunohistochemical staining for COL-II revealing strong positive expression within the CM. **(H)** GelMA after photo-crosslinking. **(I)** CM-Gel composite after photo-crosslinking. Black scale bar = 200 μm; Blue scale bar = 50 μm.

The preparation of GelMA and CM-Gel were shown in Figures 2H,I. CM-Gel exhibited a gel form appropriate for injection and demonstrated suitable viscosity, with the addition of CM enhancing the hydrogel’s viscosity (Figure 3A). Time-sweep rheological analysis further confirmed that the gel precursor underwent rapid gelation within approximately 10 s, indicating satisfactory photo-crosslinking performance (Figure 3B). The incorporation of CM significantly increased the storage modulus of the GelMA hydrogel (Figure 3C), while it did not produce a significant effect on the compressive modulus (Figure 3D). Both the Gel and CM-Gel groups maintained consistently low swelling ratios (Figure 3E), which is advantageous for *in vivo* implantation and shape retention. *In vitro* degradation assays demonstrated that all materials underwent significant degradation within the first 8 h (Figure 3F), suggesting favorable biodegradability suitable for NP regeneration. In summary, these findings confirm the successful fabrication of the CM-Gel construct, which may serve as an ideal injectable gel material for NP regeneration.

**FIGURE 3:**
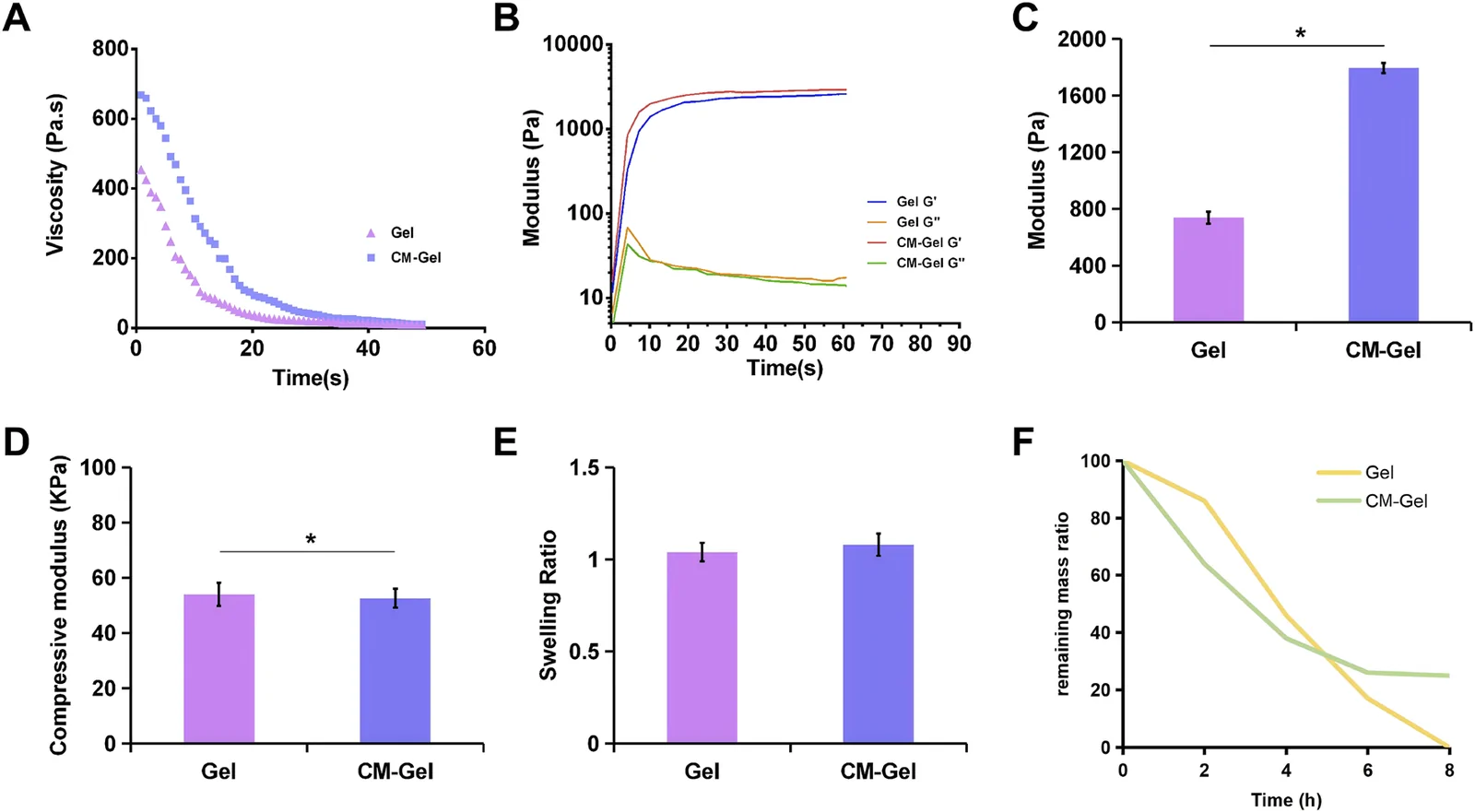
Characterization of CM-Gel. **(A)** Viscosity behavior of GelMA and CM-Gel. **(B)** Time-sweep rheological analysis illustrating gelation kinetics under UV light exposure. **(C)** Mechanical strength of Gel and CM-Gel following photo-crosslinking. **(D)** Compressive modulus of Gel and CM-Gel. **(E)** Swelling ratio of Gel and CM-Gel post photo-crosslinking. **(F)** *In vitro* degradation profiles of Gel and CM-Gel in 0.15% collagenase solution at 37 °C, with residual mass measured at 0, 2, 4, 6, and 8 h *P < 0.05.

### 3.2 Biocompatibility of CM-Gel *in vitro*

The results of the cell viability assay demonstrate that CM-Gel supported a healthy growth state during *in vitro* culture, evidenced by significant cell proliferation and a low rate of cell death on days 1, 4, and 7 (Figure 4A). Furthermore, analysis of cell proliferation indicated a gradual increase in total DNA content within the CM-Gel system over the culture period (Figure 4D). Collectively, these findings indicate that the CM-Gel composite material exhibits excellent biocompatibility and provides a conducive microenvironment for chondrocyte growth.

**FIGURE 4:**
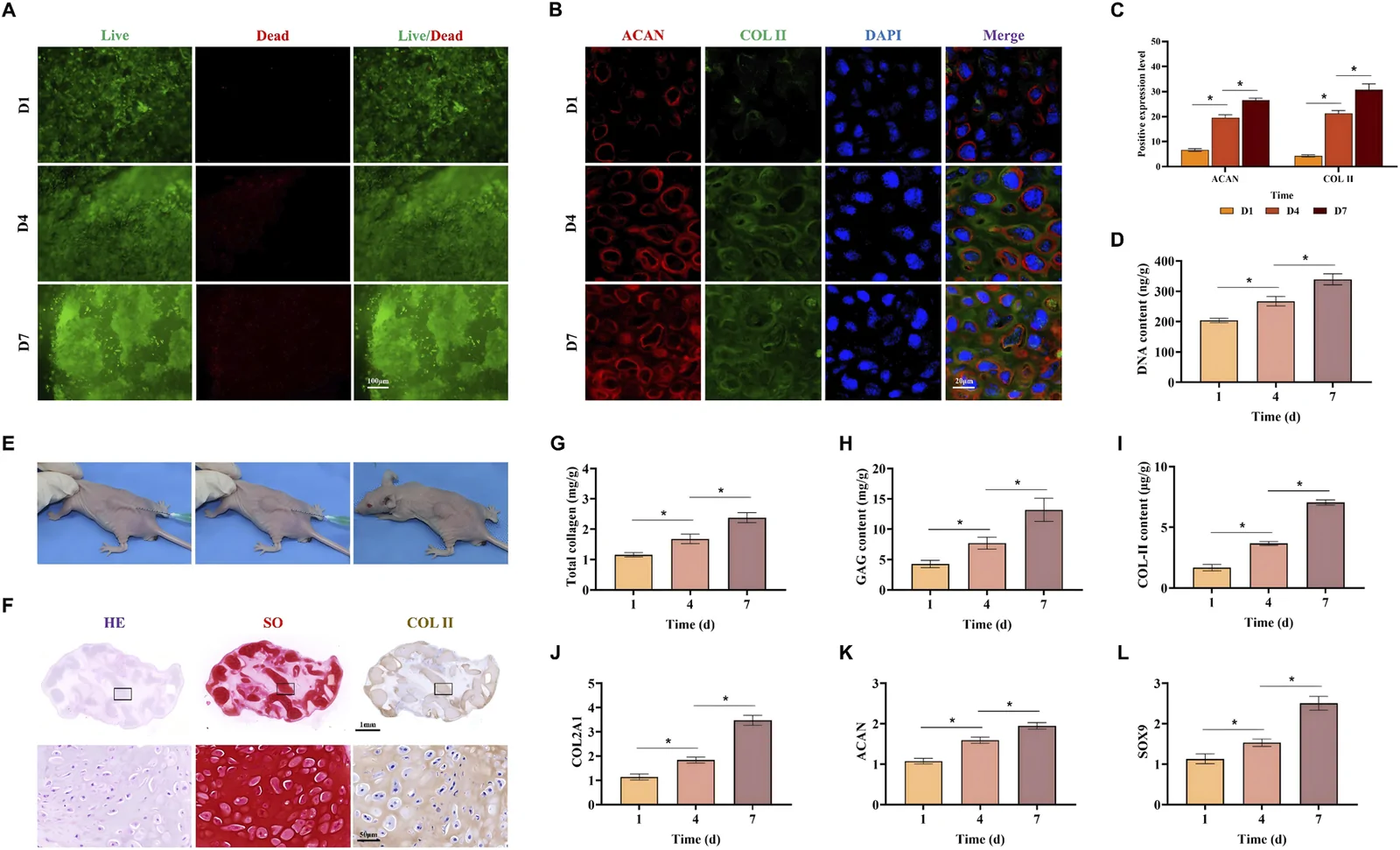
Evaluation of biocompatibility and cartilage regeneration of CM-Gel *in vitro*. **(A)** Live/dead staining of CM-Gel cultured for 1, 4, and 7 days, demonstrating favorable cell viability. **(B)** Immunofluorescence double staining for ACAN and COL-II in CM-Gel at days 1, 4, and 7, with cell nuclei counterstained using DAPI. **(C)** Semi-quantitative analysis of fluorescence intensity for ACAN and COL-II. **(D)** DNA content measurement of CM-Gel cultured for 1, 4, and 7 days, indicating sustained cell proliferation. **(E)** Illustration of the injection procedure for subcutaneous implantation of CM-Gel into nude mice. **(F)** Histological and immunohistochemical analyses of explants after 4 weeks of implantation, revealing the formation of cartilage-like tissue characterized by typical cartilage lacunae and cartilage-specific extracellular matrix distribution. **(G–I)** ELISA quantification of total collagen, GAG, and COL-II content in CM-Gel at days 1, 4, and 7. **(J–L)** Quantitative RT-PCR analysis of mRNA expression levels of cartilage formation-related genes (COL2A1, ACAN, SOX9) in CM-Gel cultured for 1, 4, and 7 days *P < 0.05.

To evaluate the maturation process of CM cultured in CM-Gel, immunofluorescence double staining was employed to examine the spatial distribution and expression levels of ACAN and COL-II on days 1, 4, and 7 (Figure 4B). On day 1, both ACAN (green fluorescence) and COL-II (red fluorescence) exhibited weak positive expression, primarily localized around the periphery of the microtissue, with minimal colocalization observed. By day 4, the fluorescence signals of both proteins were markedly increased, with a substantial expansion of positive areas and a dense reticular fluorescence pattern evident within the extracellular matrix surrounding the cells. The regions of colocalization between ACAN and COL-II also increased, suggesting the initiation of more organized matrix deposition. By day 7, the fluorescence intensity of both proteins reached its maximum, with positive staining extensively distributed throughout the entire microtissue and the surrounding hydrogel matrix, demonstrating a pronounced overlapping colocalization pattern. The extracellular matrix structure became denser and more continuous. Quantitative fluorescence intensity analysis further confirmed a time-dependent increase in the average fluorescence intensities of ACAN and COL-II, with their expression patterns showing strong correlation (Figure 4C).

To quantitatively evaluate the matrix secretion capacity and phenotypic stability of CM cultured within the CM-Gel system over time, samples were collected on days 1, 4, and 7 for ELISA and qRT-PCR analyses. ELISA results demonstrated a progressive increase in total collagen, GAG, and COL-II content in CM-Gel, reaching maximal levels by day 7 (Figures 4G–I). Correspondingly, qRT-PCR analysis revealed that the mRNA expression levels of cartilage-specific genes COL2A1, ACAN, and SOX9 were significantly upregulated on days 4 and 7 compared to day 1, exhibiting a time-dependent increase (Figures 4J–L).

These results suggest that CM-Gel supports the continuous synthesis and accumulation of cartilage-specific extracellular matrix components during *in vitro* culture, with phenotypic maturation significantly advancing over time, thereby providing a robust foundation for subsequent *in vivo* transplantation.

The feasibility of cartilage regeneration was initially evaluated using nude mice. The process of injecting CM-Gel into nude mice is shown in Figure 4E. Following 4 weeks of subcutaneous implantation, the CM-Gel effectively produced cartilage-like tissue. Histological examination demonstrated the presence of characteristic cartilage tissue within the samples, with distinctly observable cartilage lacunae. Furthermore, immunohistochemical analysis confirmed the distribution of cartilage-specific extracellular matrix components (Figure 4F).

### 3.3 Imaging evaluation of disc height

The process of IVDD modeling and subsequent injection is illustrated in Figure 5A. Based on the results of X-ray analysis for each group, it can be concluded that IVD height exhibited changes over time (Figure 5B). With the exception of the Blank group, the DHI values of the other three experimental groups demonstrated a significant decline over the observed period. Notably, the PBS group showed the greatest deviation from the Blank group at both 4 and 8 weeks, characterized by the most pronounced downward trend. In contrast, administration of Gel and CM-Gel mitigated the reduction in DHI to some extent (Figure 5C). At 4 weeks post-surgery, no significant difference in DHI was observed between these two groups. However, the CM-Gel group exhibited a markedly better preservation of IVD height at 8 weeks. The signal intensity observed in median sagittal T2-weighted MRI images reflects the water content of the nucleus pulposus tissue, with higher signal intensity indicating greater water content (Figures 5B,D). The Blank group maintained relatively high T2-weighted signal intensity, whereas the PBS group demonstrated a significant decrease, indicative of severe dehydration and the presence of “black disc” phenomena at both 4 and 8 weeks post-surgery. The signal intensity of Gel group was lower than that of the Blank group but remained significantly higher than that of the PBS group. The signal intensity of CM-Gel group was most comparable to the Blank group, with no statistically significant difference at 4 weeks. Although the signal intensity of CM-Gel group decreased relative to the Blank group at 8 weeks, it remained significantly higher than that of the other experimental groups.

**FIGURE 5:**
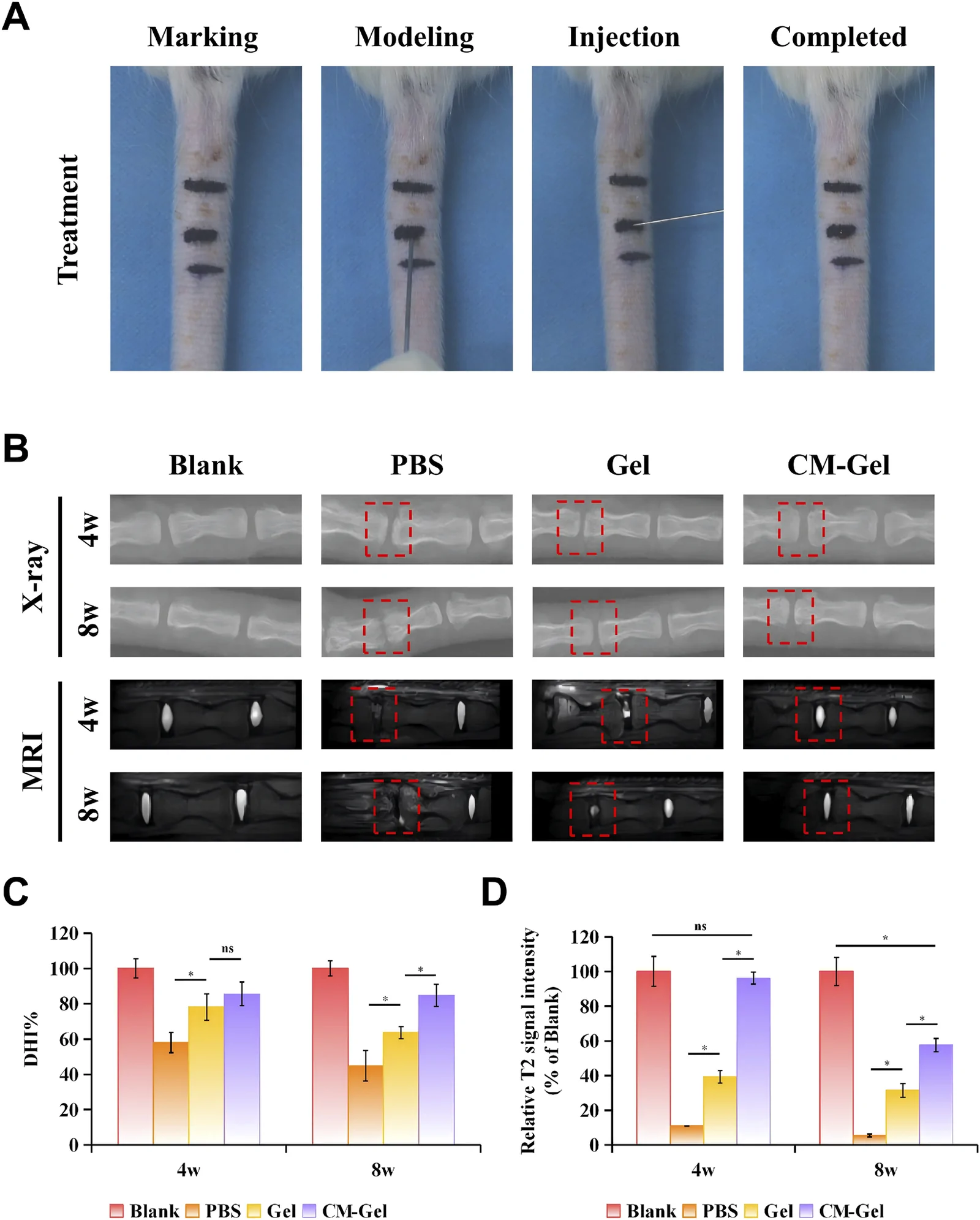
*In vivo* imaging evaluation of intervertebral disc height and hydration status. **(A)** Schematic representation of IVDD modeling and injection procedure. **(B)** Representative postoperative lateral X-ray images and T2-weighted sagittal MRI scans obtained at 4 and 8 weeks for each experimental group. **(C)** Quantitative analysis of DHI, demonstrating that CM-Gel effectively preserves intervertebral disc height. **(D)** Quantitative analysis of T2-weighted MRI signal intensity, indicative of the water content within the nucleus pulposus tissue. *P < 0.05. ns = no statistical significance.

### 3.4 Histological, immunohistochemical and immunofluorescence analysis

To further assess the therapeutic efficacy in treating IVDD, histological analyses of disc tissue were performed. HE staining was utilized to examine the morphology of the NP (Figure 6A). In the PBS group, significant loss of the NP was observed, with near-complete disappearance by 8 weeks, as the original NP region was replaced by fibrous tissue. The Gel group exhibited a gradual reduction in NP tissue over time. Conversely, the CM-Gel group demonstrated clear evidence of injection and filling at 4 weeks, accompanied by a more preserved disc structure and morphology. SO-FG staining further revealed that the central regions of the discs in both the CM-Gel and Blank groups were rich in proteoglycans. Moreover, histological scores were highest in the PBS group, reflecting the most severe degeneration, while the CM-Gel group’s scores were only marginally elevated compared to the Blank group, indicating milder degeneration (Figure 6B).

**FIGURE 6:**
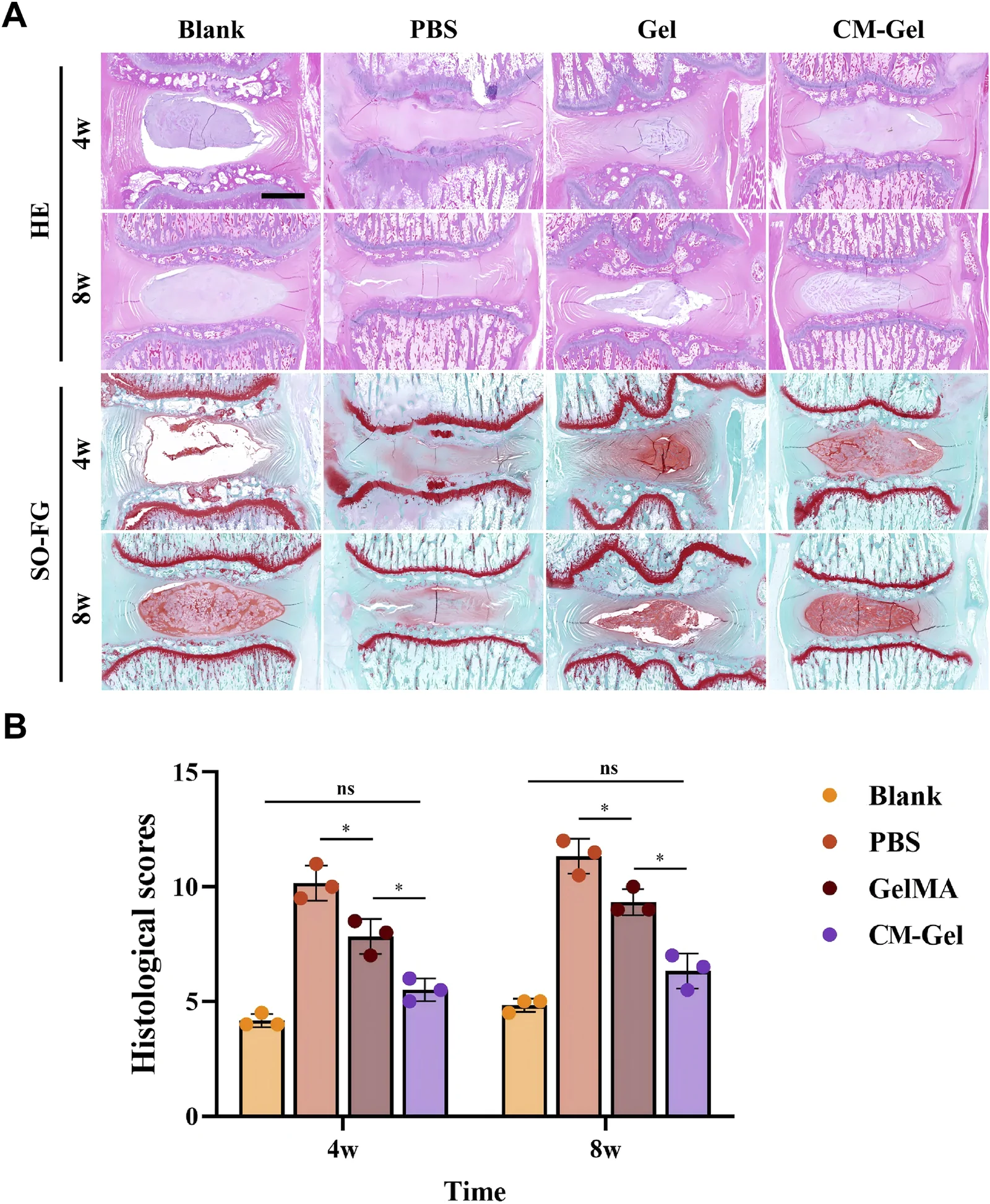
Histological analyses after treatment of IVDD *in vivo*. **(A)** Representative images of HE staining and SO-FG staining of nucleus pulposus tissue at 4 and 8 weeks post-surgery for each group. **(B)** Corresponding histological scores. Scale bar = 1 mm *P < 0.05. ns = no statistical significance.

Immunohistochemical staining for ACAN, COL-I, and COL-II was conducted to evaluate *in vivo* ECM synthesis (Figure 7A). The CM-Gel group exhibited significantly elevated expression levels of ACAN and COL-II relative to the PBS and Gel groups, whereas COL-I expression remained negative at all time points. Semi-quantitative analysis using ImageJ software demonstrated that the expression levels of ACAN and COL-II in the CM-Gel group were significantly elevated compared to those in the PBS and Gel groups at both 4 and 8 weeks. In contrast, COL-I expression remained undetectable at all time points (Figures 7B–D). To assess the regulatory impact of CM-Gel on the inflammatory microenvironment, immunofluorescence staining for TNF-α, IL-1β, and IL-6 was conducted on samples collected 8 weeks post-surgery (Figure 8). The findings indicated that, in the PBS group, cells positive for these three inflammatory markers were extensively distributed and exhibited high fluorescence intensity. Although the Gel group demonstrated a reduction relative to the PBS group, levels remained elevated compared to the Blank group. Notably, the CM-Gel group showed a significantly lower number of positive cells and reduced fluorescence intensity compared to both the PBS and Gel groups, approximating the levels observed in the Blank group. These observations were corroborated by semi-quantitative analysis. These findings indicate that CM-Gel preserves cartilage expression homeostasis and mitigates disc degeneration.

**FIGURE 7:**
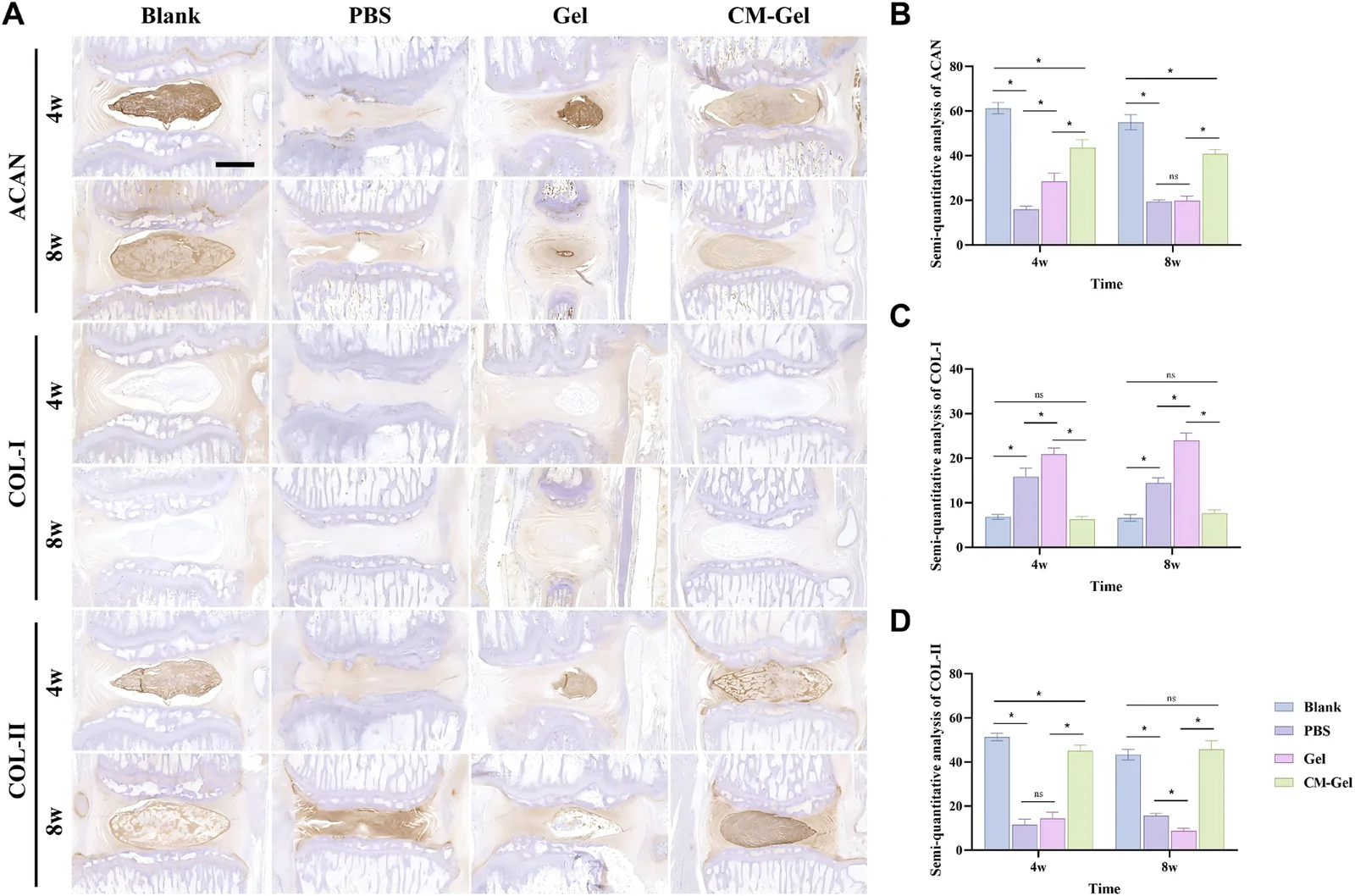
Immunohistochemical analyses after treatment of IVDD *in vivo*. **(A)** Representative immunohistochemical staining images of ACAN, COL-I, and COL-II at 4 and 8 weeks post-operation for each group. **(B–D)** Semi-quantitative analyses of ACAN **(B)**, COL-I **(C)**, and COL-II **(D)** staining. Scale bar = 1 mm *P < 0.05. ns = no statistical significance.

**FIGURE 8:**
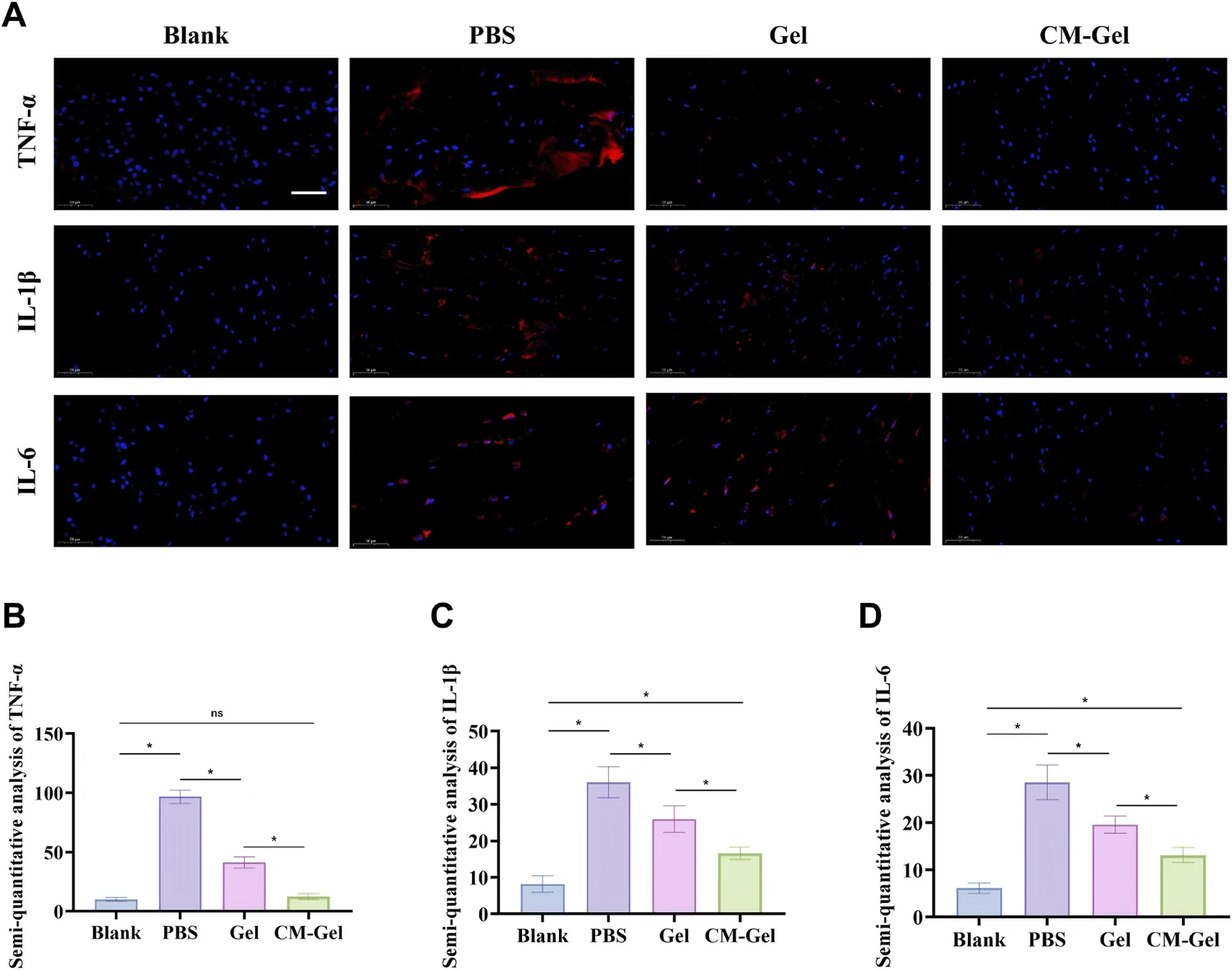
Immunofluorescence analyses after treatment of IVDD *in vivo*. **(A)** Representative immunofluorescence staining images of TNF-α, IL-1β, and IL-6 at 8 weeks post-operation for each group. **(B–D)** Semi-quantitative analyses of TNF-α **(B)**, IL-1β **(C)**, and IL-6 **(D)** staining. Scale bar = 50 μm *P < 0.05. ns = no statistical significance.

### 3.5 qRT-PCR analyses

To assess the regulatory effects of CM-Gel on the degenerative microenvironment of IVD at the molecular level, tissue samples were collected post-surgery for qRT-PCR analysis (Figure 8). Compared to the Blank and Gel groups, the mRNA expression levels of COL2A1, ACAN, and SOX9 were significantly upregulated in the CM-Gel group (Figures 9A–C). These findings suggest that CM-Gel effectively promotes the synthesis of key extracellular matrix components in nucleus pulposus cells and helps maintain the chondrocyte phenotype *in vivo*. Additionally, the expression levels of MMP13 and ADAMTS5 were significantly downregulated in the CM-Gel group relative to the other group (Figures 9D,E). Importantly, COL1A1, a critical marker of nucleus pulposus fibrosis and degeneration, was also markedly suppressed in the CM-Gel group (Figure 9F).

**FIGURE 9:**
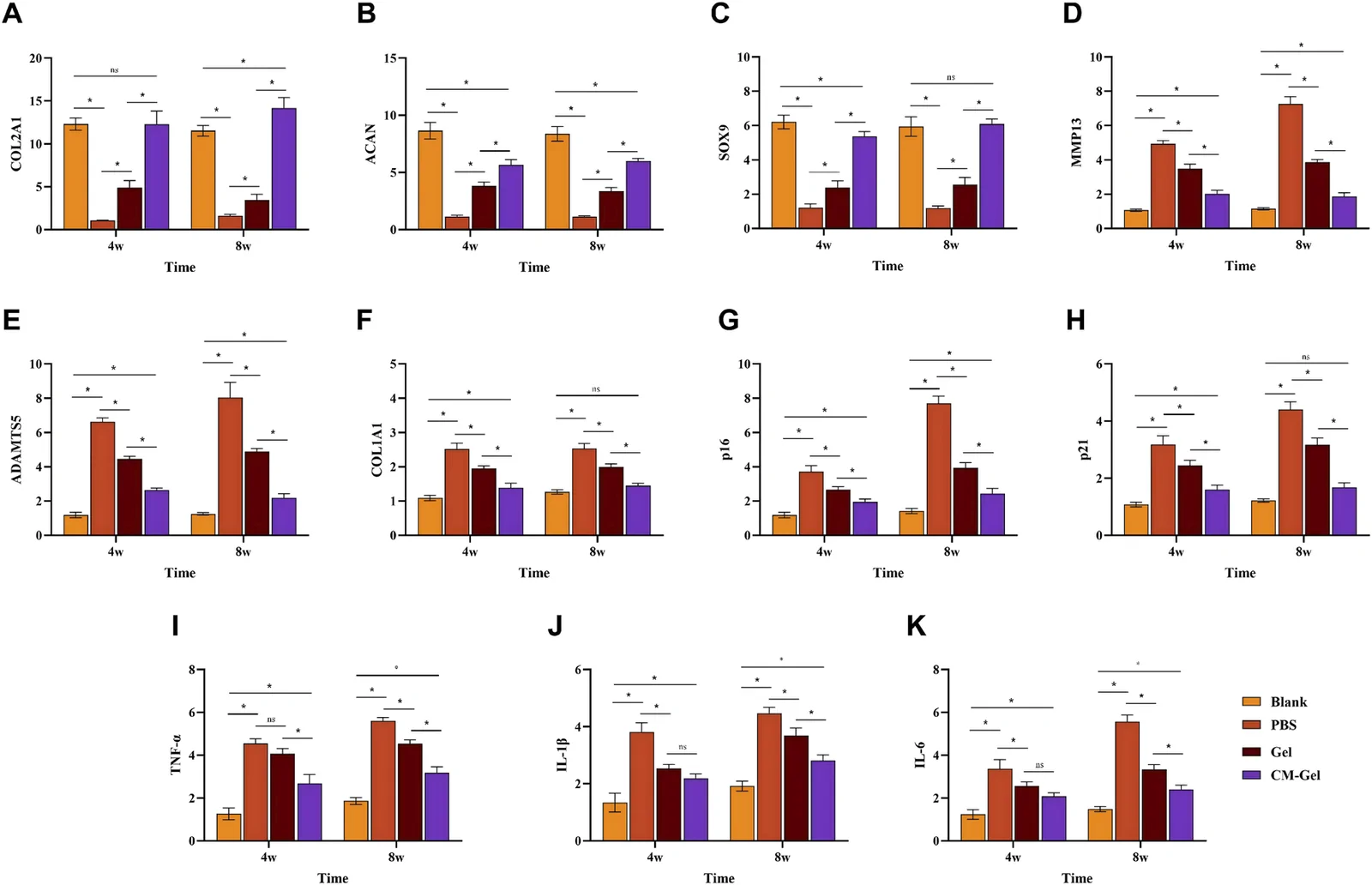
qRT-PCR analyses after treatment of IVDD *in vivo*. **(A–C)** Anabolic genes: COL2A1, ACAN, and SOX9. **(D,E)** Catabolic genes: MMP13 and ADAMTS5. **(F)** Fibrosis marker: COL1A1. **(G,H)** Senescence markers: p16 and p21. **(I–K)** Inflammatory factors: TNF-α, IL-1β, and IL-6. All samples were collected at 4 and 8 weeks post-surgery. *P < 0.05. ns = no statistical significance.

The regulatory effects of CM-Gel on nucleus pulposus cell senescence and the local inflammatory microenvironment were assessed by measuring the mRNA expression levels of senescence-associated markers (p16 and p21) and inflammatory cytokines (TNF-α, IL-1β, and IL-6) (Figures 9G–K). The results demonstrated that, relative to the PBS and Gel groups, the CM-Gel group exhibited a significant downregulation of p16 and p21 expression, indicating that CM-Gel effectively inhibits the senescence of nucleus pulposus cells. Furthermore, the expression levels of TNF-α, IL-1β, and IL-6 were markedly reduced in the CM-Gel group compared to the PBS and Gel groups, suggesting that CM-Gel ameliorates the local inflammatory microenvironment within the IVD. These findings corroborate that the therapeutic effects of CM-Gel extend beyond the promotion of ECM synthesis and metabolism to include the inhibition of cellular senescence and attenuation of local inflammation. This provides a more comprehensive molecular basis for understanding the mechanism by which CM-Gel facilitates the repair of IVDD.

In summary, the findings demonstrate that CM-Gel effectively preserves IVD height and water content of NP *in vivo*. This indicates its superior and synergistic mechanical support capabilities, as well as its ability to promote ECM synthesis, inhibit cellular senescence, and modulate the inflammatory microenvironment *in vivo*, thereby highlighting its significant potential for minimally invasive treatment of IVDD.

## 4 Discussion

This study developed and validated an injectable hydrogel derived from CM as a novel approach for IVD regeneration. This method not only effectively restores IVD height but also actively facilitates the regeneration of ECM exhibiting native nucleus pulposus-specific characteristics, thereby achieving a transition from mere “biomechanical filling” to “biofunctional regeneration”. The principal innovation of this research resides in the strategic integration of the advantages inherent to CM and hydrogels, thereby overcoming the limitations associated with their individual applications. In situ repair experiments utilizing an IVDD model, the engineered CM-Gel successfully regenerated biomimetic IVD tissue closely resembling native ECM components and maintained its structural integrity and mechanical properties for up to 8 weeks post-injection.

IVDD is characterized by the loss of mechanical function and pathological alterations in the ECM components (Liu et al., 2024; Han et al., 2023a). An effective regenerative approach must concurrently address both aspects. Previous studies have demonstrated that simple hydrogel injections can offer immediate mechanical support. However, their inherent properties restrict them to functioning solely as temporary physical fillers, thereby impeding genuine tissue regeneration (Xing et al., 2021). Additionally, the degradation rate of hydrogels is challenging to synchronize with the natural pace of tissue regeneration, resulting in premature loss of mechanical support and the continued progression of IVDD. The findings of the present study corroborate this conclusion. The Gel group exhibited effective restoration of disc height and significant mechanical improvement during the early implantation phase. Nonetheless, these benefits were transient. Imaging analyses revealed that as the hydrogel gradually degraded, its mechanical support diminished, leading to a marked reduction in disc space height and ultimately failing to arrest the degenerative process. Histological examination further indicated that the hydrogel-only group acted merely as a physical filler, with no substantial deposition of newly synthesized ECM within or surrounding the implant, thereby confirming its fundamental lack of bioactivity. It is essential to consider the cellular composition of auricular cartilage, the tissue source for CM. Recent studies have identified auricular cartilage as being abundant in lipochondrocytes (LC), a distinct cell type characterized by highly stable lipid droplets that exhibit resistance to metabolic stress (Ramos et al., 2025). Upon transfer from native tissue to a three-dimensional hydrogel culture, the altered biomechanical microenvironment may influence LC lipid metabolism and potentially affect the properties of the resulting microtissue. Nevertheless, our characterization of CM demonstrated by strong SO staining, elevated COL-II and ACAN expression suggests that, CM predominantly display a classical chondrogenic phenotype. The specific fate of LC within microtissue and their contribution to the final construct warrant further investigation in future studies.

In contrast, the CM-Gel developed in this study not only preserved the immediate mechanical support and injectability advantages characteristic of hydrogels but also conferred regenerative capabilities through the incorporation of CM. Within the CM-Gel, the hydrogel functioned not only as an injectable carrier but also provided essential physical protection to the delicate CM during the initial transplantation phase. The CM developed in this study is fundamentally a structure formed through cellular self-assembly. In contrast to the commonly observed spherical organoids, this planar configuration offers advantages including more uniform size and cell distribution. These features are particularly beneficial for standardized applications, such as integration with hydrogels and *in vivo* injection. The CM were fully encapsulated and stabilized via crosslinking, thereby compensating for their inherent high fluidity. Importantly, the hydrogel replicated the highly hydrated microenvironment of the native nucleus pulposus, serving as a biomimetic scaffold that supported the survival, proliferation, and functional maintenance of CM-Gel (Wu et al., 2022b; Han et al., 2023b). Our imaging data demonstrate that implantation of CM-Gel rapidly restores disc height, primarily attributable to the hydrogel’s immediate swelling properties and the subsequent formation of an elastic structure. Meanwhile, histological analyses demonstrated that during the early stages of IVDD, the CM-Gel actively and continuously secreted key ECM components, including COL-II and ACAN, effectively and progressively replacing the degrading hydrogel. In later stages, this process facilitated the formation of bioactive newly formed tissue, thereby achieving sustained mechanical stability. This transition from “passive support” to “active regeneration” is critical to overcoming the limitations of conventional hydrogel therapies and underscores the indispensable role of CM as the core regenerative component.

Histological analyses confirmed functional regeneration, demonstrating substantial deposition of COL-II and ACAN at the implantation site, along with seamless integration with the host tissue. These findings indicate successful preservation of the chondrogenic phenotype of CM within the challenging IVD microenvironment, contrasting with previous reports of MSC apoptosis or fibrotic differentiation under degenerative conditions (Pang et al., 2021; Dang et al., 2015). Imaging assessments demonstrated that CM-Gel exhibited superior water retention throughout the experimental period, displaying the most gradual decline among all groups. Its performance remained closest to that of the normal controls and was significantly better than that of the Gel group. The pre-formed three-dimensional structure and intercellular connections of CM likely provide a structural basis for resistance to inflammatory and hypoxic stress, thereby maintaining functional stability. qRT-PCR analysis revealed that CM-Gel significantly upregulated the expression of ACAN, COL-II, and SOX9, while downregulating MMP13, ADAMTS5, and COL-I. These findings indicate enhanced matrix synthesis, suppressed degradation, and resistance to fibrosis. Notably, SOX9, the principal transcription factor governing chondrogenesis and the maintenance of NP phenotype, may coordinate the upregulation of ACAN and COL-II, thereby indicating the reactivation of endogenous ECM synthesis through paracrine signaling. Beyond the direct secretion of ECM, paracrine mechanisms may also play a role in mediating regenerative effects. This is evidenced by the upregulation of SOX9, indicative of the activation of endogenous NP cells. Furthermore, the observed downregulation of inflammatory cytokines, including TNF-α, IL-1β, and IL-6, as well as senescence markers such as p16 and p21, suggests a modulation of the local microenvironment. Immunofluorescence staining further validated these results at the protein level, demonstrating a significantly reduced number of TNF-α-, IL-1β-, and IL-6-positive cells in the CM-Gel group compared to the PBS and Gel groups. The anti-inflammatory effects observed in CM-Gel may be attributed to the immunomodulatory functions of chondrocytes. These cells secrete anti-inflammatory factors, including IL-1Ra, TGF-β, and IL-10, which competitively inhibit the IL-1β signaling pathway (Palmer et al., 2004; Palmer et al., 2002; Wiegertjes et al., 2019; Adler et al., 2017; Elkhenany et al., 2024). Furthermore, three-dimensional cultured cartilage microtissues can suppress the activation of the NF-κB pathway in macrophages and NP cells through the secretion of soluble mediators, leading to the downregulation of TNF-α and IL-6 expression (Limagne et al., 2016; Jiang et al., 2025b; Kuppa et al., 2024). Additionally, extracellular vesicles derived from chondrocytes may transport anti-inflammatory microRNAs that modulate the inflammatory responses of host cells (Liu et al., 2023; Wang et al., 2025; Wu et al., 2025). Consequently, our strategy not only repairs the structural integrity but also restores the biological function of the tissue, offering a promising approach to reverse IVDD. Compared to existing tissue engineering methods, this study introduces novel innovations and practical advantages. Unlike composite cell-material transplantation strategies, it circumvents the open surgical trauma associated with implanting *in vitro* cultured mature composites, thereby enabling minimally invasive injection procedures. Furthermore, relative to injection therapies utilizing cells or their derivatives, this approach confers enhanced mechanical strength, functional stability, and ECM regeneration efficiency.

Although the findings are promising, several limitations must be acknowledged. First, the degradation kinetics of the hydrogel and the ECM deposition rate of microtissue require more precise synchronization. Both excessively rapid and excessively slow degradation may adversely affect the repair outcome. Second, although we successfully conducted autologous co-culture using rats chondrocytes, clinical translation remains challenged by issues related to cell procurement and donor site morbidity. Furthermore, while auricular cartilage-derived microtissue demonstrate functional efficacy, they do not entirely recapitulate the molecular complexity of native NP tissue, and the long-term risk of chondrogenic transformation unclear. Consequently, future research will focus on exploring bone marrow-derived mesenchymal stem cell (BMSC)-derived microtissue to overcome these limitations. Third, given that the CM already expressed ACAN, COL-II, and SOX9 prior to implantation, the observed positive staining and gene upregulation may partially derive from the graft itself rather than solely indicating endogenous host regeneration. Although imaging and histological data such as DHI/T2 signal maintenance at 8 weeks and graft-host integration indirectly suggest host participation, direct evidence differentiating graft-derived from host-derived contributions is absent. In future research, it will be essential to employ species-specific primers or fluorescence tracking techniques to elucidate this distinction and to monitor the distribution and leakage of the implants. Fourth, the absence of a control group comprising dispersed chondrocytes in GelMA limits the ability to draw definitive conclusions. Consequently, it remains uncertain whether the observed therapeutic effects are attributable to the microtissue-specific three-dimensional architecture or simply to the presence of viable chondrocytes and pre-deposited ECM. It is noteworthy that a scaffold-free approach, the cell sheet technology circumvents the challenge of matching hydrogel degradation with ECM deposition. The three-dimensional microtissue approach presents several distinct advantages, including pre-established cell-cell interactions, immediate ECM functionality following implantation, and the potential for minimally invasive delivery. Future studies incorporating appropriate control groups are necessary to elucidate the specific contributions of the microtissue structure.

In addition, the mechanistic insights provided remain largely descriptive, lacking direct investigation of specific signaling pathways. Although gene expression data indirectly implicate the involvement of the TGF-β/SMAD and NF-κB pathways, direct validation through techniques such as Western blot analysis or pharmacological inhibition is necessary. Furthermore, the *in vivo* assessment was confined to an 8-week observation period, which is inadequate to determine the long-term durability of the regenerative effects, particularly given the chronic progression of IVDD. Finally, the rat tail puncture model does not fully replicate the pathophysiology of human IVDD due to differences in anatomy, size, mechanical loading, and degeneration mechanisms. Therefore, studies employing large animal models with extended follow-up periods of six to 12 months are warranted to assess long-term safety and functional stability prior to clinical translation.

Beyond the scientific findings, the clinical translation of CM-Gel encounters several additional practical challenges. First, the current manual static culture system demonstrates significant batch-to-batch variability, highlighting the need to transition towards automated. Second, the quality control of living microtissue necessitates the development of a comprehensive set of critical quality attributes, as traditional physicochemical assays are inadequate for assessing biological functionality. Third, cryopreservation and transportation present considerable technical challenges, given that microtissue viability post-thaw is frequently suboptimal. Fourth, as a combined advanced therapy medicinal product, the regulatory framework for CM-Gel remains unclear and requires harmonization with evolving regulatory standards. Finally, although autologous sourcing mitigates immunogenicity concerns, patient-specific manufacturing introduces additional complexities related to standardization and cost-effectiveness. Besides, the safety of *in situ* UV photocrosslinking remains a critical consideration. In this study, 365 nm UVA light was applied at an intensity of 20 mW/cm^2^ for 5 s. Since nucleic acids exhibit maximal absorption near 260 nm, UVA photons at 365 nm do not possess sufficient energy to directly cause significant DNA damage. This was corroborated by *in vitro* Live/Dead staining and DNA quantification, which demonstrated high cell viability under the specified conditions. *In vivo*, the UV dose reaching the disc tissue is further diminished by the presence of 1–2 mm of overlying skin. Nevertheless, visible-light crosslinking systems, such as those utilizing 405 nm wavelengths, offer a potentially safer alternative and will be investigated in future research. Overcoming these translational challenges through interdisciplinary collaboration and regulatory engagement will be imperative for the successful clinical implementation of this technology.

## 5 Conclusion

In summary, this study successfully developed an active injectable hydrogel derived from CM, a material that effectively integrates the advantages of both constituents. This biomaterial presents a promising approach for the treatment of IVDD by simultaneously facilitating structural reconstruction and functional regeneration. Consequently, it introduces innovative potential to the field of IVDD and offers a novel therapeutic strategy applicable to the repair of other cartilage-related tissues.

## Data Availability

The original contributions presented in the study are included in the article/supplementary material, further inquiries can be directed to the corresponding authors.
